# Differences in left and right lower limb control strategies in coping with visual tracking tasks during bipedal standing

**DOI:** 10.3389/fspor.2024.1421881

**Published:** 2024-07-12

**Authors:** Tadayoshi Minamisawa, Noboru Chiba, Eizaburo Suzuki

**Affiliations:** ^1^ Department of Physical Therapy, Yamagata Prefectural University of Health Sciences, Yamagata, Japan; ^2^ Department of Occupational Therapy, Yamagata Prefectural University of Health Sciences, Yamagata, Japan

**Keywords:** balance control, bipedal standing, visual target tracking, sum of areas, center of pressure

## Abstract

**Introduction:**

Differences in motor control between the lower limbs may influence the risk of sports injury and recovery from rehabilitation. In this study, differences in the visual feedback ability of the left and right lower limbs were assessed using visual target tracking tasks.

**Methods:**

Thirty-four healthy young subjects (aged 20.4 ± 1.2 years) were asked to move their bodies back and forth while tracking a visual target displayed on a monitor in front of them for 30 s. The two target motions were sinusoidal (i.e., predictable patterns) and more complex (random) patterns. To assess the ability of the lower limbs to follow visual target tracking, antero-posterior CoP (right limb, CoPap–r; left limb, CoPap–l) and medio-lateral CoP (right limb, CoPml–r; left limb, CoPml–l) data were measured using a stabilometer. Tracking ability by visual feedback ability was calculated as the difference in displacement between the target signal and the trajectories of the right and left pressure centers as trapezoidal areas, and a smaller sum of area (SoA) over the entire measurement time was defined as a greater tracking ability.

**Results:**

Regarding the SoA in the anterior-posterior CoP, the mean SoA in the sinusoidal and random tasks was significantly lower in the CoP-r data than in the CoP-l data, indicating that the right lower limb had a more remarkable ability to follow visual target tracking. Regarding the SoA in the medial-lateral direction (CoP), the mean SoA in the sinusoidal and random tasks did not significantly differ between the two legs.

**Discussion:**

The right lower limb may have a tracking function activated by the target signal when responding to visual stimuli. Identifying the motor strategies of each lower limb in response to visual stimuli will not only help identify potential differences between each lower limb but also suggest the possibility of enhancing the role of each lower limb in balance control.

## Introduction

It is well known that humans preferentially use one side of the body during voluntary movements. Such lower limb dominance (or lateral dominance) may influence functional performance (1), and it has been concluded that there is an association between lower limb dominance and injury risk (2). Against this background, several authors have discussed whether balance ability differs between dominant and nondominant legs (3, 4). Previous studies on lower limb lateralization suggest that during motor control, the primary sensorimotor cortex and basal ganglia lateralize the activation patterns of the joint movements of the dominant and nondominant legs of the lower limb, thereby producing undesirable signals and filtering out desirable movement patterns (5, 6). Therefore, differences in motor control between the left and right lower limbs may manifest as differences in balance strategies (6). If a clinically important aspect is understanding how both lower limbs balance independently and in concert, it would be clinically meaningful to assess the task with a bipedal stance rather than assessing one leg independently (7, 8).

We propose introducing a visual tracking task to examine functional differences and advantages between the lower limbs. Because real-world environmental stimuli are unpredictable and complex, visual tracking task with random target motion to induce feedback control of postural changes has been proposed to assess standing balance performance to increase the demand for sensorimotor integration (9–11). Assessing the ability to track a constantly changing target, such as in a visuomotor tracking task, can provide more information about sensorimotor control than can stationary tasks (12, 13). Therefore, visuomotor tracking may help researchers investigate changes in different functional roles of the lower limb. In a previous study on the ability to track postural changes using visual cues, the modulation of the intralimb muscles in response to visual target tracking differed between the left and right lower limbs, suggesting differences in the neuromuscular strategies of the left and right lower limbs in response to visual information (14). Thus, visual target tracking stimulation tasks may reveal potential left–right differences in movement strategies (15). We also propose using the motor performance of the lower limb in a visual tracking task (tracking ability) as the outcome variable to investigate motor control characteristics. These indices have revealed different functional connections between left and right intralimb muscle pairs via intramuscular coherence analysis (14). Understanding the functional differences between the left and right lower limbs in balance performance may help evaluate and optimize athletes' ability to prevent injuries and exercise due to functional asymmetry between both legs and improve the effectiveness of treatment programs and fall prevention measures in older adults. The main objective of the current investigation was to determine whether tracking accuracy on visual tracking tasks is appropriate for characterizing differences in left and right lower limb movement control. We hypothesized that a visual tracking task could be used to measure differences in accuracy between the right and left feet, which could be used to infer foot dominance.

## Materials and methods

### Participants

G*Power (3.1.9.2, HUU, Düsseldorf, Germany) was used to perform a statistical power analysis for the required sample size. Regarding prior research, we determined that a sample size of 34 participants is necessary to obtain a power of 0.8 with an effect size of 0.5 (16) and *α* = 0.05 for the matched-pairs *t*-test.

Thirty-four healthy young adults (12 males and 22 females, age: 20.4 ± 1.2 years, height: 164.9 ± 8.2 cm, weight: 59.0 ± 9.2 kg) with no history of neuromuscular or skeletal diseases were recruited from among the university personnel. Participants self-reported their dominant foot on the Waterloo Footedness Questionnaire-Revised Version (17), with 32 participants reporting their right lower limb and 2 participants reporting their left lower limb on the kick-a-ball item. The mean weight-bearing ratios (%) of the right and left lower limbs at rest were 50.01 ± 0.03 for the right lower limb and 49.9 ± 0.03 for the left lower limb when the body weight was 100%. Written information about the study was provided to all participants, and written informed consent was obtained. The experiment was approved by the Ethics Committee of Yamagata University of Health Sciences (Approval ID: 2203-35) and conducted according to the Declaration of Helsinki.

### Measurement tasks and content

To examine functional differences between limbs, the CoP trajectories of the left and right lower limbs during visual target tracking were measured with a stabilometer. Regarding the direction of the tracking task, previous reports have shown no difference in postural tracking performance or target signal coupling (cross-approximate entropy) between the AP and ML directions (18). It has also been reported that the ability to actively track in the anteroposterior direction relative to visual target cues limits the integration of AP sway into vertical motion cues due to neuromuscular constraints. Furthermore, the importance of AP sway training in constructing intervention programs to improve perceptual behavior in aging individuals for fall prevention has been emphasized (19). Therefore, in this study, the direction of the tracking task was limited to the anteroposterior direction only. The CoP data of the right and left lower limbs included antero-posterior CoP (right limb, CoPap–r; left limb, CoPap–l) and medio-lateral CoP (right limb, CoPml–r; left limb, CoPml–l) data. Although the visual tracking task in this study consisted of voluntary movements in the anterior-posterior direction, CoP data were also collected in the medial-lateral direction to account for lateral motor control (20) due to loading and unloading in the tracking task.

**Figure 1:**
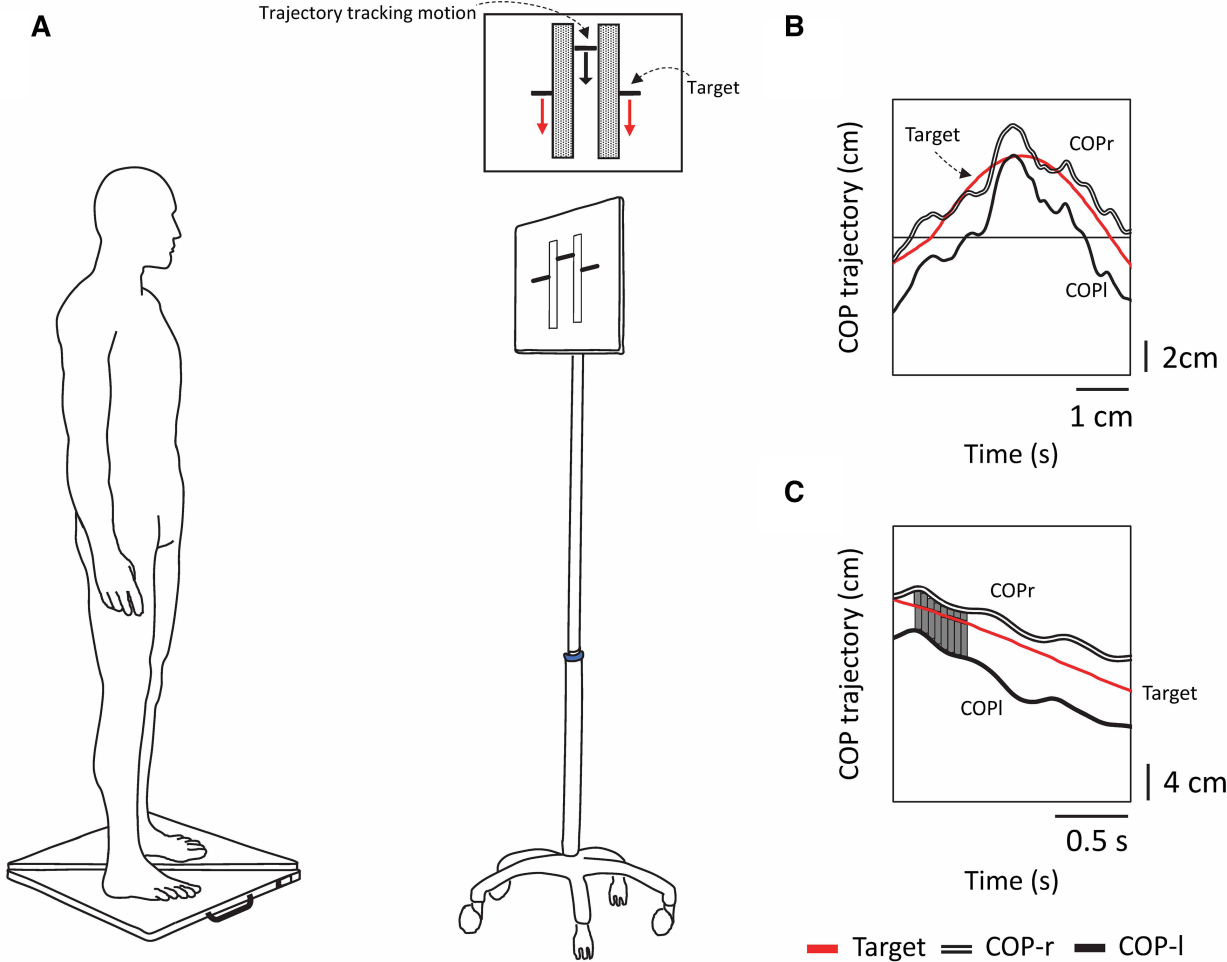
Experimental schematic and tracking tasks and analysis methods used in the experiment. (**A**) Experimental setup. Participants stood on two stabilometers and moved their bodies back and forth while tracking a vertically moving visual target. The forward motion of the CoP corresponded to the upward motion of the bar, and the backward motion of the CoP corresponded to the downward motion of the bar. Each tracking task lasted 30 s, and there were a total of two tracking tasks (periodic [sinusoidal] and acyclic [random]). (**B**) The red line shows the computer-generated target signal, the double line shows the trajectory of the tracking motion of the participant's right foot center of pressure (CoPr), and the solid line shows the trajectory of the tracking motion of the left foot center of pressure (CoPl). (**C**) The displacement between the target signal and the trajectory of CoPr and CoPl was calculated as the trapezoidal area, and the sum of the areas (SoA) was obtained over the entire measurement time.

The stabilometer consisted of three load cells, and the CoP was calculated from the vertical component of the ground reaction force. Participants were instructed to stand barefoot, keep their eyes open, keep each foot on a separate stabilometer (sampling frequency 20 Hz, G-6100, ANIMA, Japan), and maintain a comfortable arm and stance width (Figure 1). The anterior-posterior positions of the feet were marked with tape so that the same place would be used for all trials. The task and visual stimulus methods followed our previous work on visual target tracking and intermuscular coherence (14). Participants performed a tracking task in which they had to follow vertically oscillating visual targets displayed on a monitor screen (42 × 24 cm; placed 1 m in front of them) while rocking their bodies back and forth without bending their hips or knees as much as possible (Figure 1). The CoP motion displayed on the monitor was shown as a composite CoP trajectory, in which the CoPap–r and CoPap–l were synthesized. The experimenter's cue initiated the tracking task when the target bar approached the midpoint of the participant's foot length. The two tracking tasks (sinusoidal and random waveforms) were adapted from a previously published report (11). The software that generates the target signal is an optional signal program of the manufacturer of the stabilometer. The target signal generation program in the PC outputs the variation to the monitor, and at the same time, the CoP measured by the stabilometer is input to the PC wirelessly and synchronized. The digital signals from the two stabilometers were stored on a computer for further processing.

### Target motion

**Figure 2:**
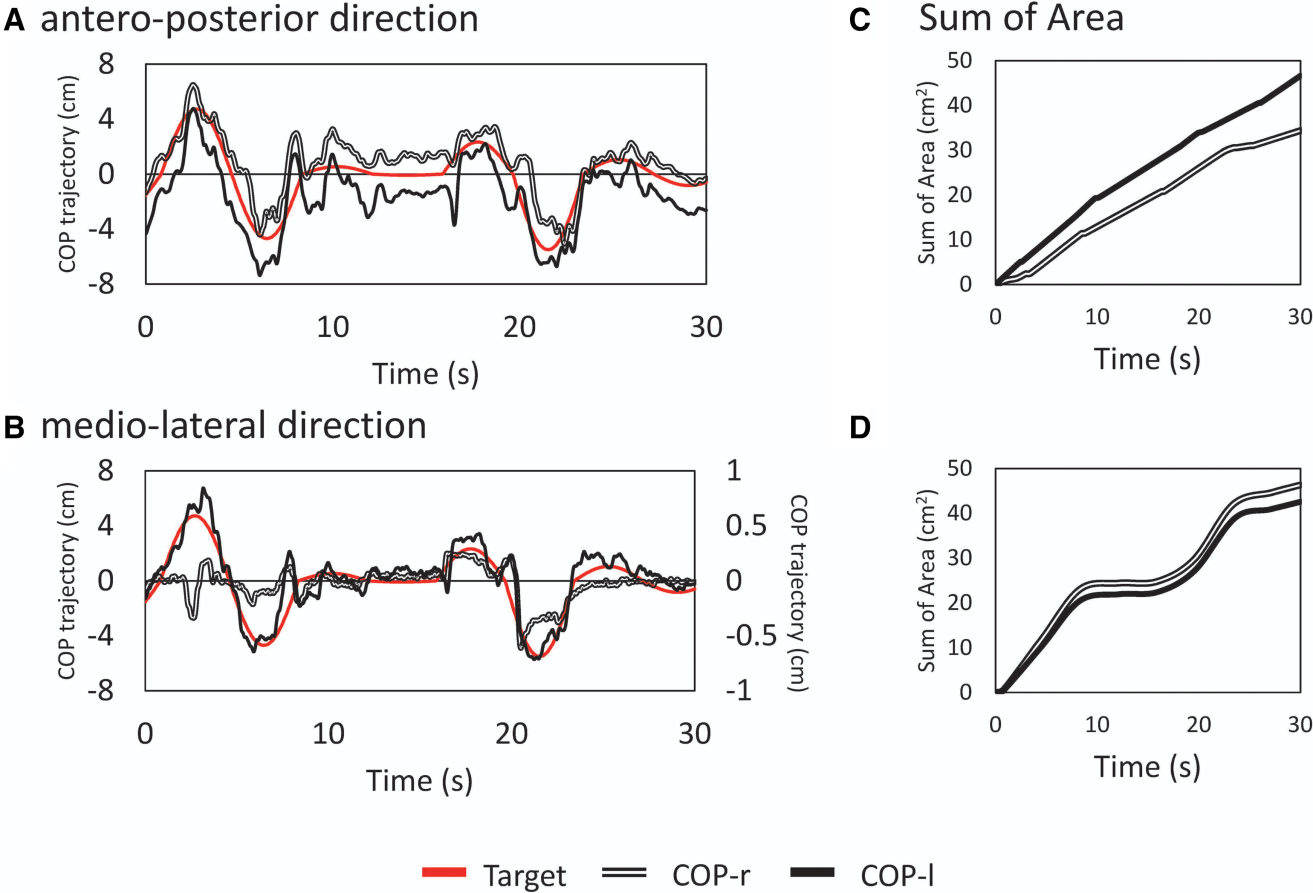
Examples of typical time series waveforms and analysis results. Typical time series in the anterior-posterior [top row; (**A**)] and mediolateral [bottom row; (**B**)] directions for a random wave tracking task. The principal axes represent the amplitudes of the target signal and center of pressure, and the abscissa represents time. Note that in (**B**), the COPml displacement is shown on the secondary axis. This is a subaxis for magnification since the displacement in the medio-lateral direction is small. The red line represents the target signal, the double line represents the displacement of tracking by the right lower limb (CoPr), and the solid line represents the displacement by the left lower limb (CoPl). The deviation between the target signal and actual CoP is shown as the sum of areas (SoA) (**C**) and (**D**) The vertical axis shows the error between the target and tracked signals as an area. A smaller value is interpreted as a greater ability to track against the target signal.

In the visual target tracking task, a bar showing the trajectory of the subject's tracking motion was displayed in the center of the screen, with a target at either end of the bar. The target was moved up and down the screen, signaling forward and backward motion. The upward movement of the target corresponded to the forward movement of the subject's CoP; the downward movement of the target corresponded to the backward movement of the subject's CoP (Figures 1A, 2A,B). The oscillation of the target movement followed the methods of a previous study (19) using a waveform with a frequency of 0.13 Hz. The target signal was programmed with a sine wave with an amplitude of ±3 cm in the anterior-posterior direction from the stabilizer reference point and a random waveform that added or subtracted another 3 cm in amplitude to the sine wave, making the fluctuations even more complex. This random waveform pattern not only fluctuated constantly but also included zero amplitudes where the target did not cue any participant movement. In addition, the fluctuation pattern differed from trial to trial, making it difficult to predict. Three trials of the two postural tasks were performed in a pseudorandomized order for six trials. Each trial lasted 30 s, with a 1-min break between trials, and a practice trial was performed before the start of the experiment.

### Data processing and analysis

The CoP signal was filtered with a fourth-order Butterworth lowpass filter with a cutoff frequency of 6 Hz. To examine the tracking of the attitude to the target signal, the displacement between the target signal and the trajectory of the right COP and left CoP was calculated as a trapezoidal area, and the sum of the areas (SoA) was obtained over the entire measurement time. If there was an intersection between the two curves (i.e., the trajectory of the target signal and the right or left COP), the coordinates of the intersection were recalculated, a triangle was formed with the points around it, and the area of this triangle was also calculated to obtain the SoA. If there was a point discrepancy in the X–coordinates (time interval) of the two curves, the trapezoidal area was calculated after generating the points by interpolation (Figure 1C). A small value of the SoA indicated a high tracking ability for the target signal. The total COP trajectory length for all subjects for the program-generated target signal was 58.54 ± 0.07 cm for the sine signal and 59.17 ± 7.34 cm for the random signal. Random signals have different trajectories for each trial; there is concern about the effect of trajectory length on the SoA (e.g., increased area due to longer trajectories); however, there was no significant correlation between the total trajectory length of random signals and the SoA (COPap–l; *r* = −0.049, COPap–r; *r* = −0.170).

### Statistical analysis

All measured data were used and analyzed to identify differences in the SoA between the lower limbs in the tasks. First, the normality of the data was tested using the Shapiro‒Wilk test (*p* > 0.05). The Mann‒Whitney *U*-test was used to compare the SoA of the right and left lower limb CoPs for visual target tracking in each task. The statistical significance level was set at *p* < 0.05. Effect sizes were expressed using Cohen's guidelines (21). The effect sizes were interpreted as small, *r* = 0.1; medium, *r* = 0.3; and large, *r* = 0.5. The data are presented as task means and standard deviations. All the statistical analyses were performed with OriginPro 2019 (OriginLab, Northampton, MA, USA).

## Results

This study examined how the left and right lower limbs cope with a visual target tracking task to clarify lower limb dominance using tracking ability as an indicator. The results showed a statistically significant difference in SoA tracking ability in the anterior-posterior direction between the sinusoidal and random visual target tracking tasks, indicating a difference in visual feedback ability between the left and right limbs.

### Comparison of the SoA between the left and right lower limb CoPs

#### SoA in the antero-posterior direction CoP

**Figure 3:**
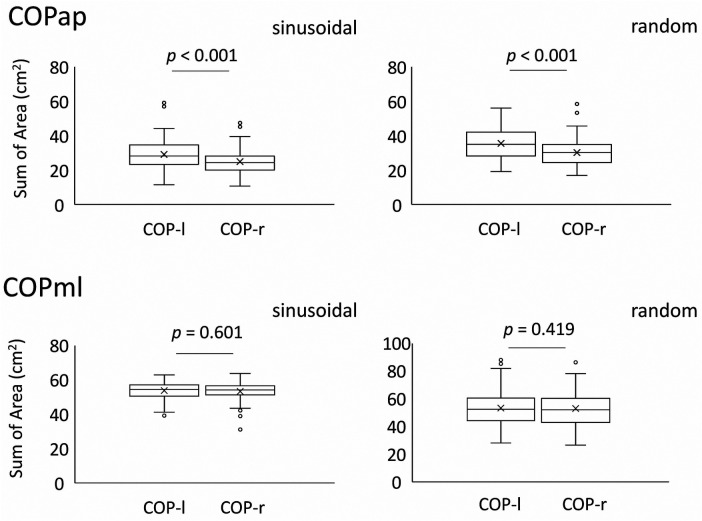
SoA of the left and right CoPs in each task compared between the two legs. The top panel shows the mean and standard deviation for all examinees in the anterior-posterior direction, and the bottom panel shows the mean and standard deviation for all examinees in the medio-lateral direction.The lower and upper whiskers of the boxplot represent the minimum value within the 1.5 interquartile range (IQR) of the first quartile, and the maximal value within the 1.5 IQR of the third quartile, respectively, and the white circles mark the outliers.

The mean SoA in the sinusoidal task was 25.10 ± 6.94 for CoPap–r and 29.40 ± 8.79 for CoPap–l (*p* < 0.001, *r* = 0.63, *U* = 6755, *z* = 3.683). The mean SoA in the random visual target tracking task was 30.21 ± 7.29 for CoPap–r and 35.73 ± 8.68 for CoPap–l (*p* < 0.001, *r* = 0.77, *U* = 7,091, *z* = 4.480). These data indicate that orbit-following performance differed between the left and right lower limbs (Figure 3, Table 1).

**Table 1:** Sum of area (SoA) of the center of pressure trajectories of the left and right lower limbs for two different target motions: The visual pursuit task consisted of a sinusoidal wave and a random waveform with complex fluctuations. The SoA in the AP is the error related to the agreement of the two curves (target signal trajectory and right or left limb) in the anterior-posterior direction over the entire measurement time, while the SoA ML is the data related to the agreement in the medio-lateral direction.

	**SoA AP direction**			**SoA ML direction**		
	**right CoP**	**left CoP**	***p* value, Effect Sizes (*r*) U value, z score**	**right CoP**	**left CoP**	***p* value, Effect Sizes (*r*) U value, z score**
sinusoidal	25.10 ± 6.94	29.40 ± 8.79	*p* < 0.001	53.80 ± 4.48	53.16 ± 4.91	*p* = 0.601
			*r* = 0.63			*r* = 0.14
			U = 6755			U = 5539
			z = 3.683			z = 0.798
random	30.21 ± 7.29	35.73 ± 8.68	*p* < 0.001	52.73 ± 12.78	53.13 ± 12.78	*p* = 0.419
			*r* = 0.77			*r* = 0.04
			U = 7091			U = 5427
			z = 4.480			z = 0.228

Values are presented in mean ± standard deviation.

#### SoA in the medial-lateral direction CoP

The mean SoA in the sinusoidal task was 53.16 ± 4.48 for CoPml–r and 53.16 ± 4.91 for the left CoPml–l (*p* = 0.601, *r* = 0.14, *U* = 5,539, *z *= 0.798). The mean SoA in the random visual target tracking task was 52.73 ± 12.78 for CoPml–r and 53.13 ± 12.78 for CoPml–l (*p* = 0.419, *r* = 0.04, *U* = 5,427, *z* = 0.228). These results indicate that the lateral sway produced when the body is voluntarily moved to cope with visual target tracking has comparable trajectory tracking performance in the left and right lower limbs (Figure 3, Table 1).

## Discussion

The purpose of this study was to determine whether left–right asymmetry in lower limb motor function is manifested by a visual tracking task during upright standing. As a result, left-right differences were observed in the ability to track the target signal. These findings contribute to understanding the previously unknown roles of the left and right lower limbs in balance strategies. These findings are discussed in further detail below.

### SoA during the visual tracking tasks

In both visual pursuit tasks (sinusoidal and random), the right COP showed a significantly lower SoA than did the left COP. This finding suggested that when controlling postural sway in response to a visual target, the right lower limb follows more precisely than does the left lower limb. When a visual tracking task exacerbates CoP-target binding, the neuromotor system modulates its response to enhance muscle modulation to ensure robust motor control to cope with the task (16). If visual target tracking disrupts visuomotor integration in the bipedal stance (10), this may be because the right foot CoP requires accurate responses to complex visuomotor cues. Typically, the ankle joint generates the torque necessary to control forward and backward sway (22, 23). Even during visual target pursuit, the plantar flexors of the ankles generate forces that slow the body's forward inertia and cause backward sway (19). Based on the SoA data in this study, we speculate that the right lower limb contributes more to tracking during target pursuit. On the other hand, the lower tracking ability of the left lower limb compared to that of the right lower limb (i.e., increased SoA values) may reflect a strategy of using muscle coactivation to stabilize movement (24). Previous studies investigating intramuscular coherence to visual target tracking have shown increased broadband modulation in the left lower limb (14). Such muscle modulation increases unilateral lower limb stiffness (14, 24), which may reduce the redundancy of lower limb joint motion in the sagittal plane. In the case of the upper limb, the cerebral hemispheres control the dynamics of the dominant hand's motor trajectory, while the nondominant limb controls limb position (4). According to Promsri et al. (25), functional asymmetry of the cerebral hemispheres is involved in motor control of the lower and upper limbs, suggesting that the control properties differ between the dominant and nondominant legs. The functional differences observed in the present study in dealing with visual target tracking may be related to such motor control theory at the cerebral level. In the case of the CoPml, no significant differences in tracking ability were found between the right and left lower limb CoPs. This may be because postural sway was limited to the anterior-posterior direction in the current task, and in general, tracking ability is always in the opposite phase due to loading and unloading in the case of control in the ML direction; therefore, similar tracking ability was obtained. Although this study did not statistically compare SoA values in different directions (i.e., CoPml vs. CoPap), the mean values were greater for CoPml than for CoPap. In the current task, the error between the trajectory of CoPap and the reference signal is suppressed because the goal is to align with the reference signal, which moves in the vertical direction by rocking the body in the anterior-posterior direction. On the other hand, the medio-lateral direction is not intended to follow the reference signal. As a result, the error in the SoA of the CoPml was more significant than that in the CoPap, which may reflect the slight weight shift of the left and right lower limbs associated with anterior-posterior movement (26).

Differences in motor control between the lower limbs are more pronounced when responding to complex visual target tracking. Previous reports suggest that aperiodic tracking tasks (i.e., random target movements) in neuromotor programs may facilitate feedback-based control, improve the internal modeling of postural motor commands, improve subsequent sensory outcomes, and prevent future falls (25). Furthermore, the current study's results indicate that the tracking task differentiates between the roles of the two legs in balance control, regardless of the task's complexity, suggesting that it also enhances role sharing. The notion that lower limb lateralization affects injury risk and recovery after rehabilitation is not new. To avoid such risks, a quantitative assessment of the role of lower limb dominance is a desirable first step toward better motor performance. For this purpose, introducing a visually guided tracking task response could be a simple and rapid assessment.

This study has several limitations. One of the most important things to note is that the relationship between the dominant foot and SoA was not analyzed in this study; only left and right SoA values were compared. Therefore, the relationship between dominant foot and tracking ability remains unclear. In addition, the subjects included not only the right lower limb dominant group but also part of the left lower limb dominant group, and the study design did not consider the dominant leg. In the future, validating the data in a sufficient sample of left-footed subjects and correlation analysis (or regression analysis) of SoA and lower limb dominance should be done, considering the above points. Second, although the subjects in this study were instructed to suppress hip and knee joint movements as much as possible to maximize ankle joint function, the possibility that the hip and knee joints were involved in stabilizing the body while performing the visual tracking task cannot be ruled out. Previous studies have shown that motor strategies with an oscillation frequency of less than 0.5 Hz are reflected in increased muscle activity in the distal muscles (20); however, this may not be sufficient to explain the results of this study in terms of ankle function alone. Third, functional differences between the left and right lower limbs were assessed regarding performance on a visual task focused on tracking performance. In addition to these parameters, the analysis of left and right lower limb reaction times to the target signal using cross-correlation function analysis as a time-domain analysis may provide a more robust description of the functional segregation between the left and right lower limbs. Fourth, the only parameter used in this study was our proposed SoA, and its superiority or inferiority to conventional indices remains to be determined. A comprehensive analysis using many parameters will provide a multifaceted understanding of future left and right lower limb dominance.

## Conclusions

The present study showed a left–right difference in tracking ability when tracking random target motion, with the right lower limb tracking the target signal with greater ability in the anterior–posterior direction. The results for right lower limb tracking ability in anteroposterior perturbations support our initial hypothesis. There was functional differentiation in the strategies of the two lower limbs by visual target tracking, which may have induced intrinsic coordination between the two limbs when the participants maintained their balance while standing. Such a paradigm could be proposed as the foundation for a rehabilitation program to assess individuals with unstable postural control and to improve individuals' limb coordination during complex movements.

## Data Availability

The raw data supporting the conclusions of this article will be made available by the authors, without undue reservation.
